# Spatially resolved metabolic analysis reveals a central role for transcriptional control in carbon allocation to wood

**DOI:** 10.1093/jxb/erx200

**Published:** 2017-06-22

**Authors:** Melissa Roach, Stéphanie Arrivault, Amir Mahboubi, Nicole Krohn, Ronan Sulpice, Mark Stitt, Totte Niittylä

**Affiliations:** 1 Umeå Plant Science Centre, Department of Forest Genetics and Plant Physiology, Swedish University of Agricultural Sciences, Umeå, Sweden; 2 Max Planck Institute for Molecular Plant Physiology, Potsdam-Golm, Germany; 3 Plant Systems Biology Laboratory, Plant AgriBiosciences Research Centre, School of Natural Science, Galway, Ireland

**Keywords:** Aspen, *Populus*, primary metabolism, wood formation

## Abstract

The contribution of transcriptional and post-transcriptional regulation to modifying carbon allocation to developing wood of trees is not well defined. To clarify the role of transcriptional regulation, the enzyme activity patterns of eight central primary metabolism enzymes across phloem, cambium, and developing wood of aspen (*Populus tremula* L.) were compared with transcript levels obtained by RNA sequencing of sequential stem sections from the same trees. Enzymes were selected on the basis of their importance in sugar metabolism and in linking primary metabolism to lignin biosynthesis. Existing enzyme assays were adapted to allow measurements from ~1 mm^3^ sections of dissected stem tissue. These experiments provided high spatial resolution of enzyme activity changes across different stages of wood development, and identified the gene transcripts probably responsible for these changes. In most cases, there was a clear positive relationship between transcripts and enzyme activity. During secondary cell wall formation, the increases in transcript levels and enzyme activities also matched with increased levels of glucose, fructose, hexose phosphates, and UDP-glucose, emphasizing an important role for transcriptional regulation in carbon allocation to developing aspen wood. These observations corroborate the efforts to increase carbon allocation to wood by engineering gene regulatory networks.

## Teaser

Central primary metabolism enzyme activities track transcript abundance during secondary cell wall formation in wood.

## Introduction

In many tree species, including those within the genus *Populus*, most of the carbon in wood is derived from sucrose imported from the photosynthetic source tissues (Turgeon, 1996). Synthesis of the main wood cell wall polymers, cellulose, hemicellulose, and lignin, is dependent on import and subsequent metabolism of sucrose. Despite the importance of sugar metabolism in developing wood in providing energy and substrates for secondary growth, our understanding of the spatial distribution of primary metabolism and cell wall precursor biosynthesis in this tissue is limited.

Wood formation in trees is initiated in the vascular cambium, which forms a cylinder of meristematic cells around the stem. Cambium cells divide, giving rise to phloem progenitors on the outside and xylem progenitors on the inside of the meristem. Wood consists of three main cell types—xylem fibres, vessels, and ray cells. During wood formation, cells undergo expansion, secondary cell wall formation, and maturation or cell death (Mellerowicz *et al.*, 2001). These different wood developmental stages can be anatomically distinguished and separated for chemical and biochemical analysis. The bulk of the cell wall polymers that form the majority of the wood biomass are synthesized during secondary cell wall formation.

Transcript profiling of wood developmental stages during this developmental sequence has revealed intricate transcriptional regulation of wood formation and allowed the selection of candidate genes associated with particular developmental stages (Hertzberg *et al.*, 2001; Schrader *et al.*, 2004; Courtois-Moreau *et al.*, 2009). Transcript level patterns of central primary metabolism enzymes suggested an important role for transcriptional regulation of carbon allocation during wood formation. However, several studies have shown that the correlation between transcript and the corresponding protein level is not always high, and that the changes in transcript levels do not necessarily reflect changes in corresponding metabolic pathways (Stitt and Gibon, 2014). In Arabidopsis, extensive comparison of whole-genome transcript data and enzyme activities during diurnal cycles showed that enzyme activity (measured in optimized conditions providing a proxy for protein abundance, Piques *et al.*, 2009) often changes more slowly and frequently independently compared with the corresponding gene transcripts (Gibon *et al.*, 2004). The agreement became stronger when longer term adjustments were analysed (Gibon *et al.*, 2004, 2006). Comparison of transcript levels, enzyme activities, and metabolites during tomato fruit development did not show clear connectivity between the transcript abundance and enzyme activities, and even less so between metabolites and enzymes (Steinhauser *et al.*, 2010). Similarly, a focused analysis of starch metabolism in Arabidopsis leaves showed that there is no consistent correlation between transcript levels, protein levels, and metabolic flux (Smith *et al.*, 2004). In developing Arabidopsis leaves, there is little relationship between diurnal changes in transcript abundance and protein abundance, although the relationship is stronger when the comparison is made across leaf developmental stages (Baerenfaller *et al.*, 2012, 2015). These observations have led to a growing realization that protein levels are also strongly regulated post-transcriptionally, for example via translational regulation (Bailey-Serres, 2013; Juntawong *et al.*, 2014) or by the regulation of protein degradation (Piques *et al.*, 2009; Yang *et al.*, 2010; Li *et al.*, 2012, 2017; Nelson *et al.*, 2014a, b).

These examples indicate an important role for post-transcriptional as well as transcriptional regulation of metabolism, and prompted us to investigate the spatial distribution of primary metabolic enzyme activities in the developing wood of aspen, and to compare the enzyme activities with the corresponding transcript level changes across the developing wood. The following enzymes were chosen based on their central position in primary metabolism and the biosynthesis of cell wall precursors.

Sucrose phosphate synthase (SPS) catalyses the first step of the sucrose synthesis pathway by converting UDP-glucose and fructose-6-phosphate to sucrose-6-phosphate, UDP, and H^+^ in a reversible reaction (Lunn and MacRae, 2003). The reaction is rendered effectively irreversible *in vivo* because the product, sucrose-6-phosphate, is hydrolysed to sucrose by sucrose phosphatase. SPS has an important role in the regulation of sucrose synthesis in both photosynthetic and non-photosynthetic tissues (Winter and Huber, 2000). Increased SPS activity has been shown to correlate with increased cellulose biosynthesis in cotton seed fibres, and secondary cell wall formation in *Zinnia* cell cultures, suggesting a role in cell wall biosynthesis (Babb and Haigler, 2001). However, no increase in SPS activity during secondary cell wall formation was observed in the developing wood of *Pinus sylvestris* (Uggla *et al.*, 2001). In *Populus canadensis* wood, SPS activity showed large seasonal variation, increasing in the autumn, staying high during the winter months, then declining during spring and staying low during summer, indicating a role in storage and dormancy metabolism in wood (Schrader and Sauter, 2002).

Sucrose synthase (SUS) catalyses the reversible conversion of sucrose to UDP-glucose and fructose. Mutant and reverse genetic studies have shown that SUS is important for carbon entry into metabolism in sink tissues of many species including potato tubers (Zrenner *et al.*, 1995) and maize seed endosperm (Chourey *et al.*, 1998). SUS has also been proposed to have a central role in providing the UDP-glucose substrate for cellulose biosynthesis (Haigler *et al.*, 2001). SUS activity in the developing wood of *P. sylvestris* was shown to increase from the primary wall to secondary cell wall biosynthesis, supporting a role in cell wall biosynthesis (Uggla *et al.*, 2001). Mutations in SUS have established a central role for two isoforms of SUS in sucrose entry into metabolism and in the conversion of sucrose to starch in developing maize seeds (Chourey *et al.*, 1998). Reverse genetics has revealed a key role for SUS in starch accumulation in potato tubers (Zrenner *et al.*, 1995), and in sucrose metabolism in developing cotton fibres (Ruan *et al.*, 2003). However, the lack of obvious growth phenotypes in quadruple *sus* mutants of Arabidopsis (Barratt *et al.*, 2009) and *Populus SUSRNAi* lines with only 4% of developing wood SUS activity compared with the wild type (Gerber *et al.*, 2014) have challenged the idea that SUS always plays a central and general role in cell wall or cellulose biosynthesis.

Sucrose can also be hydrolysed to glucose and fructose by invertase (INV). Plants typically contain various isoforms of INV located in the cell wall, the cytosol, or the vacuole. Cell wall INVs are thought to play an important role during the apoplasmic unloading of sucrose, for example during import in developing maize seeds (Chourey *et al.*, 2006) and tomato fruit (Zanor *et al.*, 2009), while vacuolar INVs are involved in the mobilization of sucrose reserves in the vacuole and the regulation of osmotic pressure during cell expansion (Wang *et al.*, 2010; Ruan *et al.*, 2012). It was recently shown that cytosolic INV plays a key role in sucrose cleavage in growing Arabidopsis and lotus leaves (Barratt *et al.*, 2009; Welham *et al.*, 2009), and in supplying carbon for cellulose biosynthesis in the developing wood of aspen (Rende *et al.*, 2017). Thus, the emerging picture is that SUS plays a key role in sucrose cleavage in some tissues and species, and cytosolic INV in others.

Regardless of the initial sucrose cleavage mechanism in developing wood, hexose phosphorylation is likely to be important for carbon entry into metabolism. Fructokinase (FRK) phosphorylates fructose to fructose-6-phosphate, and wood-expressed isoforms of FRK were shown to be important for cellulose biosynthesis in aspen wood (Roach *et al.*, 2012). Hexokinase (HXK) preferentially phosphorylates glucose to glucose-6-phosphate (Renz and Stitt, 1993), and therefore plays a central role in glycolytic energy production as well as several biosynthetic pathways including nucleotide sugar biosynthesis for cell wall biosynthesis. Reversible reactions catalysed by phosphoglucoisomerase (PGI) and phosphoglucomutase (PGM) can interconvert glucose-6-phosphate and fructose-6-phosphate and glucose-6-phosphate and glucose-1-phosphate, respectively.

The reversible reaction catalysed by UDP-glucose pyrophosphorylase (UGPase) can synthesize UDP-glucose from glucose-1-phosphate and UTP, providing an alternative UDP-glucose synthesis route to SUS (Kleczkowski *et al.*, 2004). When sucrose is degraded via SUS, UGPase can further metabolize UDP-glucose produced by SUS in excess of that required for cell wall synthesis. In this context, it is interesting that the total extractable UGPase activity was an order of magnitude higher than SUS activity in the developing wood of 6-week-old hybrid aspen (*Populus tremula*×*tremuloides*) (Roach *et al.*, 2012). The spatial distribution of UGPase activity in developing wood has not been investigated.

The synthesis of the aromatic amino acid phenylalanine from sucrose is critical for lignin biosynthesis, but the location of this pathway in relation to wood development has not been established. Lignin precursors, the monolignols, are derived from phenylalanine (Boerjan *et al.*, 2003). Phenylalanine is derived from the shikimate pathway, which converts erythrose 4-phosphate (E4P) and phospho*enol*pyruvate (PEP) into aromatic amino acids, linking primary and secondary metabolism (Herrmann and Weaver, 1999). Experiments with *Pinus taeda* cell cultures revealed that addition of phenylalanine to the cell culture led to a concomitant increase in monolignol biosynthesis and a change in the monolignol ratio, indicating that carbon allocation to phenylalanine was rate limiting for lignin biosynthesis (Anterola *et al.*, 2002). In hybrid aspen (*Populus sieboldii*×*Populus grandidentata*), immunolocalization revealed that the first shikimate pathway enzyme 3-deoxy-d-*arabino*-heptulosonate 7-phosphate synthase (DAHPS), which catalyses the condensation of PEP and E4P to DAHP, co-localized in the same differentiating xylem fibre cells as several lignin pathway enzymes (Sato *et al.*, 2009). This observation suggested that carbon from primary carbohydrate metabolism is allocated to lignin biosynthesis in the lignifying cells (Sato *et al.*, 2009). Shikimate dehydrogenase (SHDH) is the fourth enzyme in the shikimate pathway converting 3-dehydroshikimate to shikimate in a reversible NADPH-consuming reaction (Herrmann and Weaver, 1999). Increased SHDH activity was shown to correlate with increased lignin biosynthesis after exposing hybrid aspen (*Populus tremula*×*alba*) leaves to ozone (Cabané *et al.*, 2004). Six-week-old greenhouse-grown hybrid aspen (*Populus tremula*×*tremuloides*) trees showed high SHDH activity in developing wood (Roach *et al.*, 2012). Hence, understanding the spatial distribution of SHDH activity in developing wood would inform where the primary sugar metabolism connects to the lignin biosynthesis pathway.

We have investigated the spatial distribution of several enzyme activities from central primary metabolism and related sugar pools as well as SHDH activity across the phloem, cambium, and developing wood (cell expansion, secondary cell wall formation, and maturation/cell death zones) of *Populus tremula*. The samples were collected from 45-year-old trees undergoing active wood formation. The results showed clearly that both SUS and UGPase activities increase during secondary cell wall formation and that this is closely followed by a pronounced increase in SHDH activity. Most of the other assayed primary metabolism enzyme activities also increased during secondary cell wall formation. The results indicate that both SUS and UGPase could contribute to the UDP-glucose production for secondary cell wall biosynthesis and that the carbon flux from sugars to lignin monomers takes place at the site of active lignification. Importantly, the activities of several of the assayed enzymes tracked the level of corresponding gene transcript(s), indicating that in developing wood transcriptional regulation plays a central role in determining carbon allocation.

## Materials and methods

### Experimental design of the developing wood enzyme and sugar analysis

Stem samples were harvested at midday from four ~45-year-old aspen (*Populus tremula*) trees in the middle of the growing season in early July at Mullkälen, Sweden. Trees were cut with a chain saw, and stem pieces (~3 cm×6 cm×1.5 cm; width×length×thickness) were frozen in liquid nitrogen. Phloem, cambium, and the developing wood were sectioned into 20 μm thick sections from frozen stem tissue blocks (block dimensions 13–18 mm×10 mm×2–4 mm) using a microtome (Microm HM505E, GMI Inc., Minnesota, USA). The volume of each section was estimated from the section dimensions.

### Sampling and pooling scheme

**Fig. 1.**
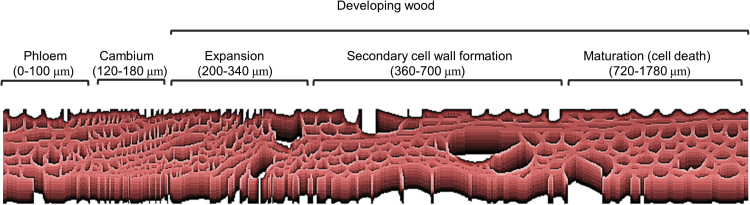
Location of the cryosection samples in phloem and developing wood including cambium, expansion, secondary cell wall formation, and maturation (cell death) zones. Numbers below each developmental zone indicate the distances of the zone from the first phloem section in micrometres.

Serial sectioning was conducted starting at the active phloem region and continuing through the cambium and to the mature xylem (Fig. 1; Supplementary Table S1 at *JXB* online). These serial sections were used to determine enzyme activities and metabolite quantities across the entire gradient of wood development. In most cases, experimental samples correspond to single microtome sections in order to provide the highest possible resolution across the developing wood zone, but in some cases it was necessary for samples to be pooled (Supplementary Table S1). Supplementary Table S1 also shows the location. Pooling of samples was conducted to decrease the number of samples when multiple sections represented similar developmental stages (active phloem, mature xylem), and to ensure sufficient protein content for accurate enzyme activity measurements (mature xylem). Experimental samples for the section series were as follows: one experimental sample representing the active phloem, which consisted of two pooled sections. Single sections were used to provide one experimental sample in the cambium, three experimental samples across the xylem expansion zone, and six experimental samples across the secondary cell wall formation zone. Three experimental samples spanned the xylem maturation zone, each sample consisting of a pool of six sections. Uneven pooling of samples was corrected for by normalization against the combined section volumes, and the data are presented as units or amounts per mm^3^ of tissue. For pooled samples, the average distance of pooled sections from the phloem is shown.

### Protein extraction for enzyme assays

Extracts were prepared at 4 °C by homogenizing sections in extraction buffer containing a final concentration of 50 mM HEPES/KOH, pH 7.5, 10 mM MgCl_2_, 1 mM EDTA, 1 mM EGTA, 1 mM benzamidine, 1 mM ɛ-aminocapronic acid, 250 μM DTT, 1 mM phenylmethylsulphonyl fluoride, 0.1% (v/v) Triton-X-100, 20% (v/v) glycerol, and polyvinyl polypyrrolidone (Gibon *et al.*, 2004; Sulpice *et al.*, 2010). In all cases, samples were extracted in 100–600 μl of extraction buffer to allow for 100-fold (v/v) dilution of the initial sample volume. For example, experimental samples consisting of one section (volume 0.8–1.3 mm^3^) were extracted in 100 μl of extraction buffer; experimental samples consisting of six pooled sections were extracted in 600 μl of extraction buffer. Extracts were either used directly in enzyme assays or subaliquoted and diluted to a final sample volume to extraction volume ratio of 200-fold (v/v). Final dilutions of extracts for assays were determined on a per enzyme basis during the validation stage of the experiment and were based on previous optimization of the enzyme assays (Gibon *et al.*, 2004).

### Continuous enzyme assays

PGI and PGM activities were measured continuously by monitoring NADP^+^ reduction produced via coupling reactions catalysed by glucose-6-phosphate dehydrogenase (G6PDH) activity. PGI activity was measured as described previously (Jones *et al.*, 1986; Cross *et al.*, 2006). PGM activities were measured as described previously by Gibon *et al.* (2009).

In short, the reaction mixes included 5 μl of the 200-fold (w/v) dilution of the initial extracts. The assay mixes contained final concentrations of 10 mM MgCl_2_, 0.1 M Tricine/KOH, pH 8, 2 mM EDTA, 0.5% (w/v) BSA, 0.05% (w/v) Triton X-100, 1.2 mM NADP, and 0.1 U of G6PDH grade II. The reactions were started by adding 2 mM fructose-6-phosphate or 3 mM glucose-1-phosphate for PGI or PGM, respectively. Assays were conducted in 96-well microplates in total volumes of 100 μl. Reactions were incubated at 25 °C and absorbance was read at 340 nm until the reaction rate stabilized. Activity calculations for the enzymes were based on the extinction coefficient for NADP^+^ reduction.

### Cycling enzyme assays

A set of high-throughput, highly sensitive enzyme assays were previously developed using a robotic-based platform that coupled enzyme activities to a set of secondary cycling reactions (Gibon *et al.*, 2004). SPS, UGPase, and SUS activities were detected by coupling enzyme activity to a glycerol-3-phosphate cycling assay. FRK, glucokinase (GLK), and SHDH activities were detected by coupling enzyme activity to an NADP^+^ cycling assay. The cycling assay determination steps for all the above enzymes were performed as described in Gibon *et al.* (2004). For cycling assays, 2 μl of the 200-fold (w/v) extract dilution was incubated in each respective reaction mix for 20 min at 25 °C prior to conducting the cycling assay determination step. The only exception to this was the SUS activity assay, which used 2 μl of the 100-fold extract dilution and an incubation time of 40 min (Steinhauser *et al.*, 2010). SPS was assayed in the forward direction as previously described in Gibon *et al.* (2004).

Extracts and standards were added to reaction mixes containing 50 mM HEPES/KOH, pH 7.5, 10 mM MgCl_2_, 1 mM EDTA, 0.05% Triton X-100, 1 U ml^–1^ UMP-kinase, 2.5 U ml^–1^ glycerokinase, 10 mM UDP-glucose, 50 μM ADP, and 50 μM dihydroxyacetone-P (DAP). Enzyme activity was assayed under *V*_max_ conditions by adding 12 mM frucose-6-phosphate and 36 mM glucose-6-phosphate. UDP produced by SPS activity was quantified spectrophotometrically by an NADP^+^ cycling assay at 340 nm.

UGPase activity was assayed in the direction of UDP-glucose breakdown. The assay was described previously (Keurentjes *et al.*, 2008; Steinhauser *et al.*, 2010).

Extracts and standards were added to reaction mixes containing 0.1 M Tricine/KOH, pH 8, 4 mM MgCl_2_, 1.5 mM NaF, 0.05% Triton X-100, 1 U ml^–1^ glycerokinase, 5 mM UDP-glucose, and 2 mM PPi. UTP produced by UGPase activity was quantified spectrophotometrically by an NADP^+^ cycling assay at 340 nm. SUS activity was assayed in the direction of sucrose breakdown. The assay was previously described in Keurentjes *et al.* (2008) and Steinhauser *et al.* (2010).

Extracts and standards were incubated for 40 min in reaction mixes containing 0.1 M HEPES/KOH pH 7.5, 20 mM MgCl_2_, 0.05% Triton X-100, 50 mM UDP, and 100 mM sucrose. UDP-Glc produced by SUS activity was quantified spectrophotometrically by a NADP^+^ cycling assay at 340 nm.

FRK and GLK activity were assayed individually by providing the reactions with 2 mM fructose or 2 mM glucose, respectively. Assay mixes contained 0.1 M Tricine/KOH pH 8, 5 mM MgCl_2_, 0.05% Triton X-100, 0.5 mM NADP^+^, 1 U ml^–1^ G6PDH, 1 U ml^–1^ PGI (for FRK activity only), and 0.8 mM ATP. Fructose-6-phosphate and glucose-6-phosphate produced by FRK and GLK, respectively were quantified spectrophotometrically by NADP^+^ cycling assay as previously described in Gibon *et al.* (2004).

SHDH activity determination was adapted from Lourenco and Neves (1984) and was measured as described in Gibon *et al.* (2004). Extracts and standards were incubated in reaction mixes containing 0.1 M Tricine/KOH pH 8, 0.05% Triton X-100, 2 mM shikimate, and 0.5 mM NADP^+^. NADPH produced by SHDH activity was quantified spectrophotometrically by an NADP^+^ cycling assay at 340 nm.

The linear range of each enzyme assay was established using a developing wood extract dilution series, and the possible presence of wood extract-derived inhibitors was tested by comparing Arabidopsis leaf extract with a combined developing wood and Arabidopsis leaf extract.

### Metabolite quantification

Soluble sugars were extracted and quantified as previously described (Stitt *et al.*, 1989; Gibon *et al.*, 2002). In short, samples were serially extracted in hot ethanol. Glucose, fructose, and sucrose contents were successively determined in each extract by enzyme-based spectrophotometric assay of NADP^+^ reduction at 340 nm. To profile sugar phosphates, samples were prepared by chloroform/methanol extraction for reverse-phase liquid chromatography coupled to tandem mass spectrometry (LC-MS/MS) analysis, as previously described by Arrivault *et al.* (2009). Briefly, samples were extracted for 2 h at −20 °C in 3:7 chloroform/methanol. Samples were then extracted three times with ice-cold water, and the aqueous layers were removed, pooled, and evaporated to dryness in a lyophilizer, and re-suspended in water. Metabolite quantification by LC-MS/MS was performed, adding internal standards ([2,2-^2^H_2_]G6P, [1,6-^13^C_2_]F6P, [U-^13^C]G1P, and [^13^C_9_,^15^N_2_]UDPG) to the extracts after sample extraction (Arrivault *et al.*, 2015).

### Transcript analysis

Transcript data were obtained from the Aspwood database at http://aspwood.popgenie.org/aspwood-v3.0/ (Sundell *et al.*, 2016). The Aspwood database contains transcriptome data obtained by RNA sequencing (RNAseq) from cryosectioned phloem, cambium, and developing wood of the same trees harvested at the same time as samples used in this study. The transcript cluster analysis was performed using the mean values of the four biological replicate trees using the heatmap.2 function in R (v3.3.0; R Core Team, 2015). For details of the transcript level determination and associated computational approaches, see Sundell *et al.* (2016).

## Results and Discussion

### Experimental design of the enzyme activity and metabolite analysis across aspen stem

Samples were separately collected from four 45-year-old aspen (*Populus tremula*) trees. Trees were cut with a chain saw and stem pieces frozen in liquid nitrogen. Anatomical inspection and viability staining of the wood showed that all trees were undergoing active wood formation (Supplementary Fig. S1). The living developing wood zone formed an ~1300 μm thick layer when measured from the cambium. The total width of the annual ring at the time of harvest was 1600–2150 μm. Phloem, cambium, and the developing wood were sectioned into 20 μm thick sections from frozen stem blocks using a microtome. The volume of each section was estimated from the section dimensions and used to normalize the enzyme activity and metabolite data. The location of the sections in relation to stem anatomy and wood development is shown in Supplementary Table S1. The width and distance of sampling zones in relation to phloem is illustrated in Fig. 1.

### Spatial distribution of eight primary metabolism enzyme activities and corresponding transcripts

Optimized enzyme activity assays provide quantitative data and a proxy for the corresponding protein amounts (Gibon *et al.*, 2004; Piques *et al.*, 2009; Stitt and Gibon, 2014). We adapted the assays developed for Arabidopsis leaf extracts by Gibon *et al.* (2004) for phloem, cambium, and developing wood samples. The high sensitivity of the determination methods allowed us to work with strong dilution of the plant material in the enzyme assays. It was possible to measure the activity of seven central primary metabolism enzymes (SPS, SUS, UGPase, HXK, FRK, PGM, PGI, and SHDH) in 0.8–1.3 mm^3^ of tissue. INV measurements were not successful due to very low extractable activities of both acidic and neutral INVs in the cryosectioned samples. We also mined the publicly available aspen RNAseq data obtained from the same trees (Sundell *et al.*, 2016) to investigate whether the measured changes in enzyme activities may correlate with similar changes in the corresponding gene transcripts during wood formation.

**Fig. 2.**
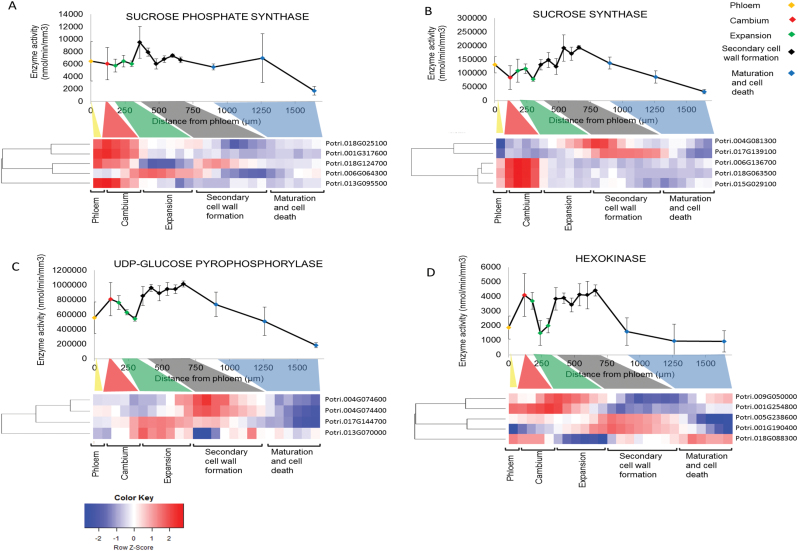

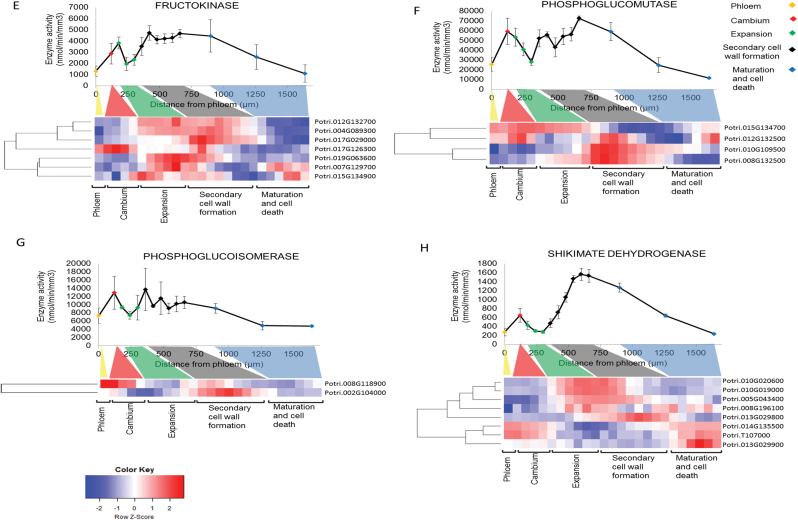
Enzyme activity and transcript levels of carbon metabolism enzymes in developing wood. Sucrose phosphate synthase (A), sucrose synthase (B), UDP-glucose pyrophosphorylase (C), hexokinase (D), fructokinase (E), phosphoglucomutase (F), phosphoglucoisomerase (G), and shikimate dehydrogenase (H). Error bars in enzyme activity plots represent the SE of four biological replicates. Transcript data are derived from http://aspwood.popgenie.org/aspwood-v3.0/. The cluster analysis was performed using the mean values of four biological replicates using the heatmap.2 function in R. Bins coloured in red in the heatmaps are significantly up-regulated and the bins coloured in blue are significantly down-regulated.

The activity of SPS was relatively constant across the stem until the last section of the wood maturation and cell death zone, where it decreased (Fig. 2A). RNAseq data showed expression of five *SPS* isoforms across the stem. Phloem and cambium showed the highest transcript levels with four expressed *SPS* genes (Fig. 2A). In developing wood, one isoform (Potri.006G064300) was expressed during cell expansion and primary wall biosynthesis, while another (Potri.018G124700) overlapped with secondary cell wall biosynthesis (Fig. 2A). This suggested SPS isoform specialization for different wood developmental stages and identified the isoforms that could contribute to the SPS activity (Fig. 2A). The steady SPS activity across the developing wood is consistent with some *de novo* sucrose synthesis during active wood formation. However, no clear increase in SPS activity was observed during secondary cell wall formation, arguing against an important role for SPS in carbon flux to cell walls at this stage.

SUS activity was relatively uniform across the phloem, cambium, and expansion zones, and showed a clear trend of increased activity across the zone of secondary cell wall formation, with the activity in the later part of this zone being 2-fold higher than in the cambium or expansion zone (Fig. 2B) After reaching a maximum at the end of the secondary cell wall-forming zone, SUS activity then began to decrease steadily towards the end of the maturation and cell death zone (Fig. 2B). Three *SUS* isoforms (Potri.018G063500, Potri.006G136700, and Potri.015G029100) were expressed in phloem and cambium, while the increase in SUS activity during wood formation corresponded to an increase in the levels of two other *SUS* isoforms (Potri.004G081300 and Potri.017G139100) (Fig. 2B). Similar to SPS isoform expression, one SUS isoform (Potri.004G081300) was induced during cell expansion and primary wall biosynthesis while another (Potri.017G139100) coincided with secondary cell wall formation. The rise in SUS activity and expression patterns of SUS isoforms across developing wood are consistent with the effects seen in transgenic *SUSRNAi* trees with almost absent SUS activity in wood (Gerber *et al.*, 2014). The *SUSRNAi* used in the study of Gerber *et al.* (2014) targeted the two wood-expressed SUSs and caused a decrease in wood density and a corresponding decrease in all the main cell wall polymers per volume of wood.

The activity of UGPase was high across phloem and cambium, and tended to decline across the expansion zone, and rise at the beginning of the secondary cell wall formation, and then remained elevated until the beginning of the cell death and maturation zone, decreasing thereafter (Fig. 2C). UGPase activity and transcript levels did not correlate well in the phloem and cambium (Fig. 2C). The increase in UGPase activity during secondary cell wall formation followed the increase in the UGPase transcripts Potri.004G074600 and Potri.004G074400 (Fig. 2C). Based on these results, it is clear that both SUS and UGPase could contribute to the UDP-glucose production during wood formation. The increase in UGPase activity was more pronounced at the beginning of the secondary cell formation while SUS activity increases more gradually. This pattern would be consistent with a central role for UGPase in providing UDP-glucose for cellulose biosynthesis, a model also supported by the recent observation of Rende *et al.* (2017) showing that the reduction of cytosolic INV activity during secondary cell wall formation reduced cellulose biosynthesis. Glucose and fructose produced by INV activity could enter the UDP-glucose pool via hexose phosphates and UGPase. So far there are no reports on the effect of reduced UGPase activity in trees. In Arabidopsis, a *ugp1ugp2* double mutant with only 6.2% of wild-type UGPase activity compared with the wild type showed severe growth phenotypes and male sterility (Park *et al.*, 2010). The reduced growth of *ugp1ugp2* plants correlated with a reduction in UDP-sugars, leading Park *et al.* (2010) to suggest that the reduction in cell wall biosynthesis precursors was the cause of the reduced growth phenotype. The severe phenotype of *ugp1ugp2* Arabidopsis suggests that a strategy reducing UGPase activity specifically during secondary cell wall formation is needed to address its role during wood formation.

Glucose-phosphorylating (HXK) activity was high in the cambium and the beginning of the cell expansion zone, was low in the middle and late part of the expansion zone, and increased early in the secondary cell wall formation zone. After this, HXK activity decreased rapidly to basal levels in the beginning of the maturation and cell death zone (Fig. 2D). Fructose-phosphorylating FRK activity showed a very similar pattern and level of activity to HXK; only the decrease in activity in the maturation zone was slightly more gradual compared with HXK (Fig. 2E). PGM catalyses the reversible interconversion of glucose-6-phosphate and glucose-1-phosphate. PGM activity also showed a bimodal activity peak, with the activity being high in the cambium, decreasing across the expansion zone, and then increasing until the end of secondary cell wall formation, after which it rapidly declined (Fig. 2F). PGI catalyses the reversible interconversion of glucose-6-phosphate and fructose-6-phosphate. PGI activity also showed a decline between the cambium and the later expanding zone, but the increase in the cell wall formation zone was less marked and not maintained in the later parts of the zone (Fig. 2G).

The activities of HXK, FRK, PGM, and PGI are all closely linked to the hexose phosphate pool, and, apart from PGI, these enzymes showed a similar pattern of activity across developing wood. FRK2 isoforms have been shown to be important for wood cellulose biosynthesis (Roach *et al.*, 2012), but the role of the other enzymes in developing wood has not been studied. At the transcript level, *HXK* was represented by five, *FRK* by seven, and *PGM* by four transcripts, all showing varied expression profiles across developing wood, with some forms being highly expressed in the phloem, cambium, and expansion zones, and others in the secondary cell wall formation zone (Fig. 2D–F). PGI activity was encoded by two transcripts, one peaking in the phloem and another during secondary cell wall formation in wood (Fig. 2G).

SHDH activity which links primary metabolism to aromatic amino acid and lignin biosynthesis had a small peak in the cambial region, was lower in the xylem expansion zone, and increased steadily to a main peak towards the end of the secondary cell wall-forming zone (Fig. 2H). The cambial peak coincides with active protein biosynthesis in dividing cells that will require aromatic amino acids, and the high activity during lignification supports a central role for SHDH in allocation of carbon to lignin. This result also suggested that phenylalanine biosynthesis takes place in the secondary cell wall-forming zone instead of being transported there from other parts of the tree or stem. The *SHDH* transcript analysis identified two transcripts in the phloem and cambium, and five transcripts in the wood coinciding with the respective increases in SHDH activity (Fig. 2H).

Whilst phloem development was not a focus of this study, we noted that there was an increase in SUS activity between the cambium and phloem (Fig. 2B) while SPS activity showed no change and UGPase, HXK, FRK, PGM, PGI, and SHDH activities all decreased. While the increase in SUS activity was not significant on a mm^3^ basis, the change in the ratios between SUS and the various hexose phosphate-metabolizing enzymes or between SUS and SHDH was significant (data not shown). This increase in SUS activity in the phloem is consistent with the idea that SUS plays an important role in metabolism within the phloem (Geigenberger *et al.*, 1993) and contributes to the sieve plate callose biosynthesis (Barratt *et al.*, 2009).

Correlations with transcript levels and enzyme activity especially during secondary cell wall formation supported a central role for transcriptional control in regulating carbon allocation to wood. For example, for *UGPase* and *SUS*, it was possible to find corresponding transcripts, which followed the enzyme activity profile. This suggested that UDP-glucose production in wood is at least partially under transcriptional control. As discussed in the Introduction, this is in contrast to several other studies in plants where such a correlation has not been observed. One reason for the poor overlap between transcript levels and enzyme activities may be that in many studies the analysed samples have contained a complex mixture of different cell types and tissues. In our study, the analysed tissues were from the same tissue and similar developmental stage, possibly resulting in better correlation of transcripts and enzyme activities. Another reason may be that transcriptional regulation plays a more important role in determining protein abundance in developmental gradients than during dynamic responses to a continually changing environment. In earlier studies, correlations were especially poor in studies of diurnal cycles. These relatively short time intervals do not allow time for changes in the abundance of many proteins that have turnover times in the order of days (Piques *et al.*, 2009; Li et al., 2012, 2017; Nelson et al., 2014a, b). Studies over longer time intervals, such as adjustment over days to new environmental conditons (Gibon *et al.*, 2004, 2006) or along leaf developmental gradients (Baerenfaller *et al.*, 2012), found a better correlation of transcript abundance with enzyme activity or protein abundance. The slow turnover of many proteins involved in central metabolism (Piques *et al.*, 2009; Schwanhäusser *et al.*, 2011; Li *et al.*, 2012) means that they do not show rapid changes in their abundance in response to short-term fluctuations in transcript levels, but will respond to longer term and sustained changes across developmental gradients (Stitt and Gibon, 2014).

### Concentrations of soluble neutral sugars and sugar phosphates across the aspen stem

**Fig. 3.**
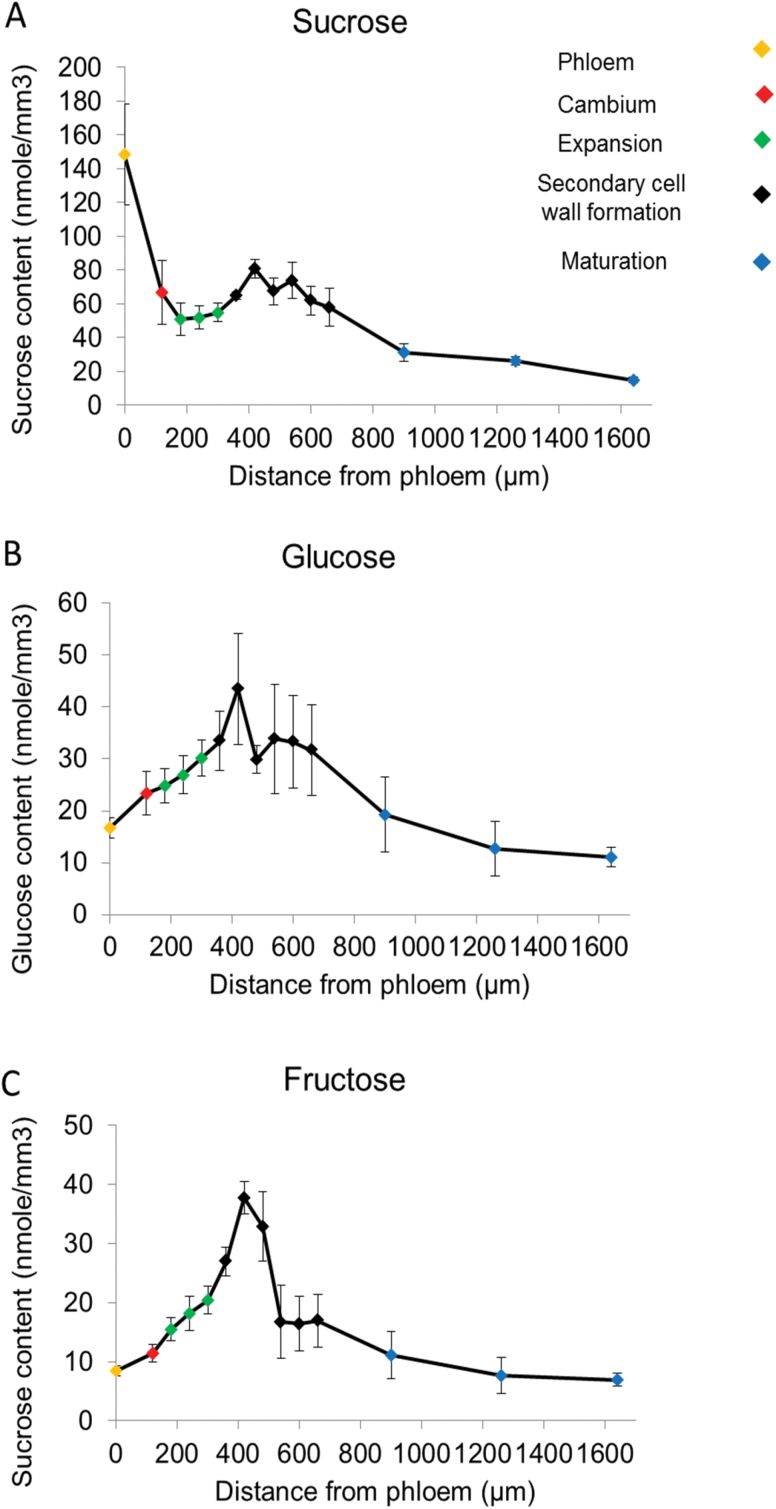
Analysis of sucrose, glucose, and fructose content in phloem and developing wood. Sucrose (A), glucose (B), and fructose (C). Error bars represent the SE of four biological replicates.

**Fig. 4.**
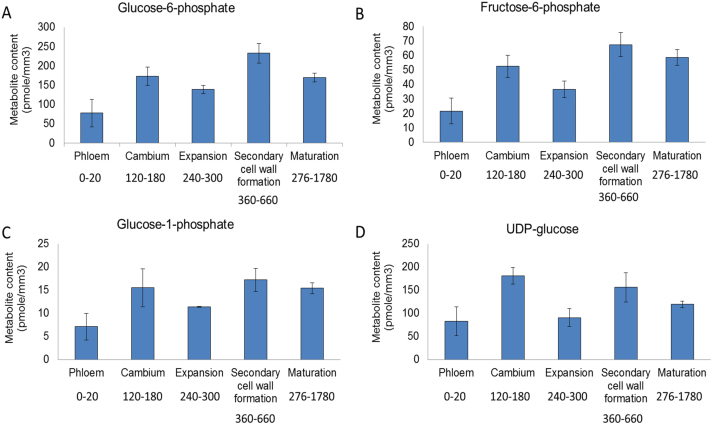
Analysis of hexose phosphate and UDP-glucose content in phloem and developing wood. Glucose-6-phosphate (A), fructose-6-phosphate (B), glucose-1-phosphate (C), and UDP-glucose (D). Error bars represent the SE of four biological replicates. Numbers below each development zone indicate the distance of the pooled sections from the first phloem section in micrometres.

Sucrose, glucose, and fructose content was measured in the phloem, cambium, and across the developing wood transect to investigate the concentration of these central sugars during secondary growth. The sucrose level was very high in the phloem, as expected. It decreased sharply in the cambium, showed a slight increase in the early part of the secondary cell wall-forming zone, and then decreased strongly again in the maturation and cell death zone (Fig. 3A). These data are in accordance with previous observations from the developing wood of *Pinus sylvestris* (Uggla *et al.*, 2001). Both glucose and fructose levels were low in the phloem, increased steadily through the expansion zone to the middle of the secondary cell wall-forming zone, and decreased in the maturation and cell death zone (Fig. 3B, C). When sucrose and the reducing sugars are compared, they showed opposite responses between the cambium and the phloem, with sucrose rising strongly and reducing sugars falling. They showed rather similar responses from the cambium through the xylem expansion, secondary wall formation and the maturation zones were rather similar, except that the peaks of glucose and especially fructose in the early part of the secondary cell wall formation zone were more marked than that of sucrose. Glucose-1-phosphate, glucose-6-phosphate, fructose-6-phosphate, and UDP-glucose showed a bimodal peak pattern with a peak in the cambial region, lower levels in the expansion zone, and a second peak in the secondary cell wall formation zone (Fig. 4). Interestingly, these intermediary metabolites remained quite high in the maturation zone possibly as a result of active salvage processes and the metabolically active ray cells, which remain alive across several annual rings.

The gradual rise in sucrose, glucose, and fructose from the cambium through the expansion zone and first part of the secondary cell wall formation zone has similarities to the increase in SUS and UGPase activities. The peak of hexose phosphates and UGPase in the cambium, the decrease in the expansion zone, and the second peak in the secondary cell wall formation zone resembles the bimodal response of HXK, FRK, PGI, and PGM activities. These peaks of hexose phosphates and hexose phosphate-metabolizing enzymes coincide with the high metabolic activity during cell division in the cambium and during secondary cell wall formation, and are consistent with there being a lower metabolic activity per tissue volume in the expansion zone. Interestingly, the small continued rise of SUS activity and the large increase in SHDH activity in the later part of the secondary cell wall formation zone occurred independently of changes in sugars, which showed a slight decline. This indicated that the rise in SHDH activity plays an important role in diverting incoming carbon towards lignin formation, and may even start to deplete sugars as they move from the phloem through the wood development zones.

### Conclusions

Comparison of changes in enzyme activities and corresponding transcript levels can provide information on whether the enzymes are mainly regulated at the transcriptional or post-transcriptional level. We adapted a set of enzyme assay protocols to determine the enzyme activities in ~1 mm^3^ of tissue corresponding to specific stem tissues and wood developmental stages. These experiments revealed a clear positive relationship between increasing activities of the key primary metabolism enzymes and increased carbon allocation to cell walls during secondary cell wall formation. In several cases, the changes in enzyme activity matched with similar changes in transcript levels in developing wood, suggesting that developmentally regulated transcription plays an important role in regulating carbon allocation to wood. In all cases, this was due to a change in expression of specific members of the gene familes that encode the enzymes. Furthermore, the changes in enzyme activities and metabolites pointed to an important role for SUS and UGPase in sucrose use and UDP-glucose biosynthesis during wood formation, for SHDH in allocating carbon to lignin formation, and for HXK, FRK, PGM, and PGI in energy metabolism. These data provide justification for the efforts to increase carbon allocation to wood by engineering gene regulatory networks.

## Supplementary data

Supplementary data are available at *JXB* online.

Fig. S1. Cross-section of aspen stem tissue.

Table S1. Location of the cryosections in the aspen stem tissue.

## Supplementary Material

Supplementary Table S1

Supplementary Figure S1

## Abbreviations:

DAHPS: 3-deoxy-d-*arabino*-heptulosonate 7-phosphate synthase
E4P: erythose-4-phosphate
FRK: fructokinase
HXK: hexokinase
INV: invertase
PEP: phospho*enol*pyrvuate
PGI: phosphoglucoisomerase
PGM: phosphoglucomutase
SHDH: shikimate dehydrogenase
SPS: sucrose phosphate synthase
SUS: sucrose synthase
UGPase: UDP-glucose pyrophosphorylase.
