# Corrigendum: Spatially resolved metabolic analysis reveals a central role for transcriptional control in carbon allocation to wood

**DOI:** 10.1093/jxb/ery202

**Published:** 2018-06-08

**Authors:** Melissa Roach, Stéphanie Arrivault, Amir Mahboubi, Nicole Krohn, Ronan Sulpice, Mark Stitt, Totte Niittylä

**Affiliations:** 1 Umeå Plant Science Centre, Department of Forest Genetics and Plant Physiology, Swedish University of Agricultural Sciences, Umeå, Sweden; 2 Max Planck Institute for Molecular Plant Physiology, Potsdam-Golm, Germany; 3 Plant Systems Biology Laboratory, Plant AgriBiosciences Research Centre, School of Natural Science, Galway, Ireland

*Journal of Experimental Botany*, Vol. 68, No. 13 pp. 3529–3539, 2017; doi:10.1093/jxb/erx200

The original manuscript contained errors in some of the figures used.

In figure 2B the gene IDs were assigned to the wrong expression pattern in the original heatmap. In figure 2H the expression pattern for the gene ID Potri.013G029900 was incorrect.

**Figure 2.**
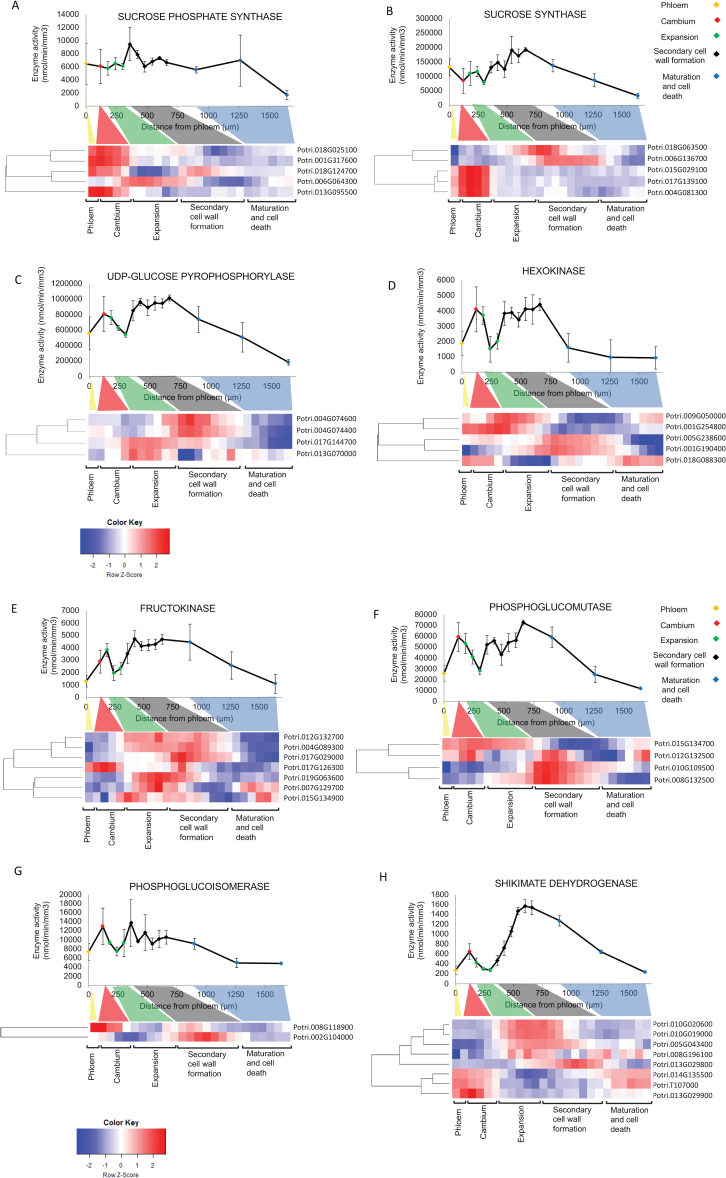


These figures have now been updated with corrected versions.

