# Supplementary material for: Spatially resolved metabolic analysis reveals a central role for transcriptional control in carbon allocation to wood
Source: J Exp Bot. 2017 Jun 22;68(13):3529–39. doi: 10.1093/jxb/erx200 (PMC5853372; doi:10.1093/jxb/erx200)
Supplement: Supplementary Figure S1 [file erx200_suppl_supplementary_figure_s1.pdf]

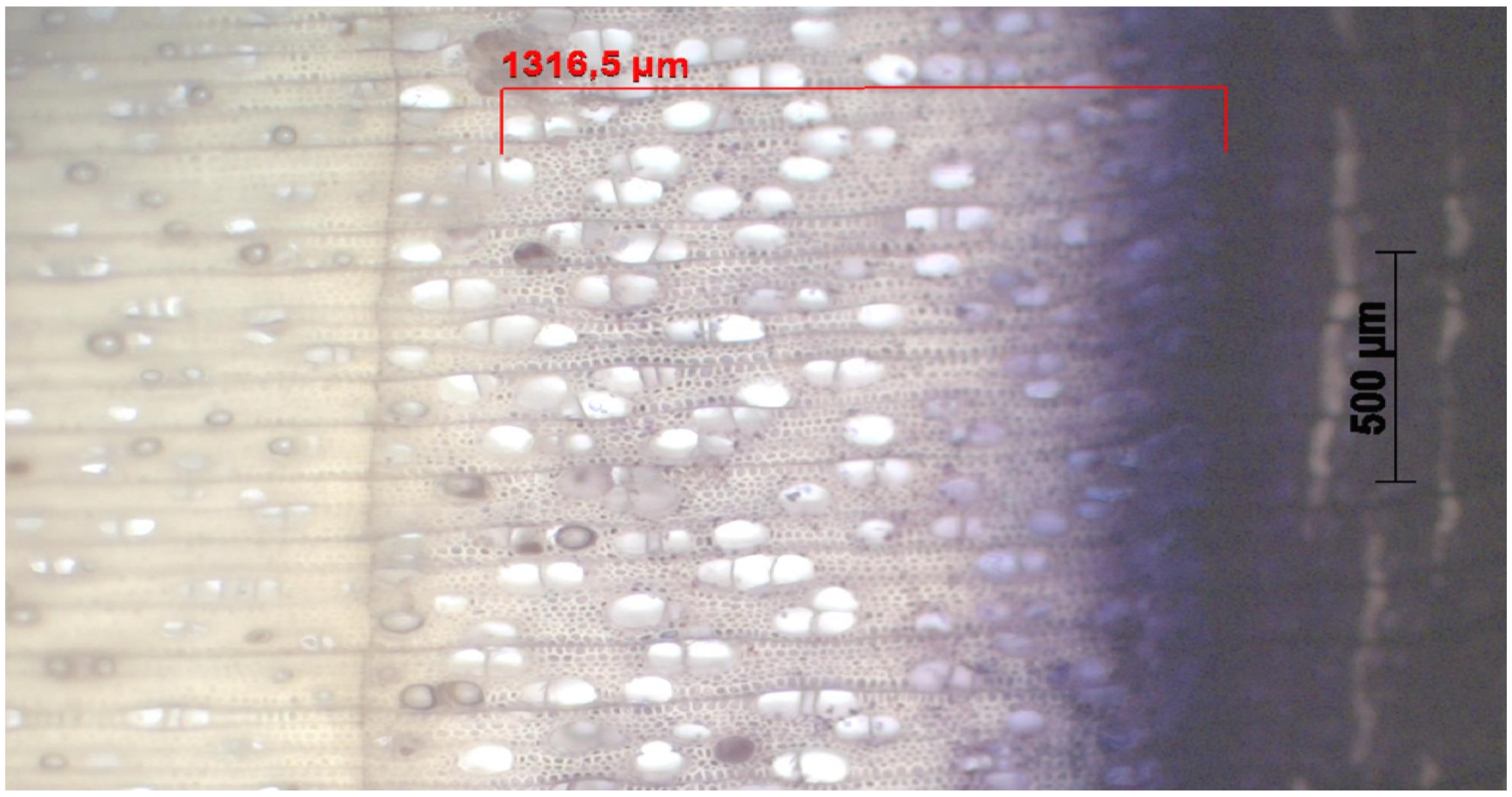

Figure S1. Example of a nitroblue tetrazolium (NBT) stained stem cross-section of the hybrid aspen trees used for enzyme activity assay. The approximate length of the living part of the wood is measured and marked in red.
